# Identification of rs28362336 as a risk SNP for generalized myasthenia gravis and its impact on HCG27 regulation

**DOI:** 10.1371/journal.pone.0359069

**Published:** 2026-09-23

**Authors:** Zihong Chen, Qingling Guo, Liting Tian, Jingnan Jin, Xia Wang, Chunyu Yu, Yichen Li, Jingjing Zhang, Yan Li, Yufan Zhao, Jiayi Zhang, Lanxin Zhang, Lukuan Qiao, Jianjian Wang

**Affiliations:** Department of Neurology, The Second Affiliated Hospital of Harbin Medical University, Harbin, Heilongjiang, China; Shuguang Hospital Affiliated to Shanghai University of Traditional Chinese Medicine, CHINA

## Abstract

Myasthenia gravis (MG) is an antibody-mediated autoimmune disease of the neuromuscular junctions. Accumulating amounts of evidence have indicated that single nucleotide polymorphisms (SNPs) and long noncoding RNAs (lncRNAs) play critical roles in the pathogenesis of MG; however, research on MG-related lncRNA SNPs remains limited. Therefore, our study focused on MG-related lncRNA SNPs to explore their association with genetic susceptibility to MG. We screened regulatory SNPs using MG GWAS data and conducted a case‒control study including 161 patients and 161 controls. After SNaPshot genotyping, LASSO regression and random forest were used to identify SNP-related risk factors. We performed eQTL analysis and ChIP-PCR to identify the functional role and underlying mechanism of rs28362336. qRT-PCR was performed to measure HCG27 expression in MG patients and to further assess HCG27 expression in generalized MG (gMG) patients. Finally, fluorescence in situ hybridization was used to determine the subcellular localization of HCG27 in CD4 + T cells, and the effects of HCG27 overexpression or knockdown were evaluated by Western blot analysis and CCK-8 assays. Significant SNPs were identified on chromosomes 1, 6, 11, 17, and 18. Although no differences in genotype or allele frequency were observed between the cases and controls overall, subtype analysis revealed that HCG27 rs9263872(TT), rs9263875(GG), and rs28362336(GG) were associated with gMG risk. The rs9263875 G allele was associated with gMG, whereas the HCG9 rs3823381 T allele was associated with ocular MG (oMG). Machine learning analysis further revealed that rs28362336 is a risk factor of MG. Bioinformatics and ChIP-PCR revealed H3K4me1, H3K27ac, and Pol II enrichment at the rs28362336 locus, suggesting enhancer activity upstream of HCG27. HCG27 expression was increased in MG patients, particularly in gMG harboring the rs28362336 GG genotype. Finally, HCG27 promoted CD4 + T cell proliferation and inhibited apoptosis. This study revealed the associations of HCG27-related SNPs with gMG and the potential regulatory role of rs28362336 in HCG27 expression. Moreover, the upregulation of HCG27 in MG patients and its proliferation-promoting effect in CD4 + T cells suggest that HCG27 may contribute to MG pathogenesis.

## Introduction

Myasthenia gravis (MG) is a chronic autoimmune disorder of neuromuscular junctions that is clinically characterized by fatigable muscle weakness [1]. As the disease progresses, MG can lead to life-threatening exacerbations, notably myasthenic crisis. MG is a rare and complex condition triggered by the intricate interplay between genetic and environmental factors [2]. The underlying genetic pathogenesis is evidenced by the high disease concordance among identical twins [3]. While associations with the major histocompatibility complex (MHC) locus have been recognized for more than 30 years, recent genome-wide association studies (GWAS) have identified numerous susceptibility variants, most of which reside in noncoding regions [4,5]. These discoveries have revealed the complex genetic landscape of MG; however, the precise links between these variants and molecular pathogenesis remain elusive because of the inherent heterogeneity of MG.

Increasing evidence suggests that many disease-associated variants identified by GWAS exert their pathogenic effects by modulating gene expression. Specifically, risk SNPs in noncoding regions are highly enriched in cis-regulatory elements, such as enhancers, which are characterized by specific histone modifications [4]. These “enhancer SNPs” can alter the binding affinity of transcription factors (TFs) in an allele-specific manner, thereby driving the dysregulation of downstream target genes [6–8]. Among these targets, long noncoding RNAs (lncRNAs) have emerged as critical mediators of immune homeostasis [9]. Although lncRNA SNPs have been increasingly implicated in autoimmune diseases such as systemic lupus erythematosus (SLE) and rheumatoid arthritis [10,11], their systematic involvement in the regulatory landscape of MG remains poorly understood [12,13].

Within the genetic architecture of MG, the MHC on chromosome 6p21.3 is the most critical susceptibility locus [14,15]. This region is dense with not only HLA protein-coding genes but also numerous noncoding elements whose functions are only beginning to be decoded. Among these, HCG27 and HCG9 are two prominent lncRNAs situated within this high-risk cluster. While HCG27 has been linked to immune dysregulation in SLE patients [16], its expression and pathogenic role in MG patients remain largely unexplored. Crucially, the clinical transition from ocular MG (oMG) to generalized MG (gMG) suggests a divergent molecular basis that may be governed by specific genetic variants within these MHC-located lncRNAs [17–19]. However, systematic investigations into how these lncRNA SNPs contribute to the immunopathological heterogeneity of MG subtypes are currently lacking.

To bridge this gap, we employed an integrative approach combining large-scale GWAS dataset screening with SNaPshot minisequencing to identify functional lncRNA SNPs in an MG cohort. We used machine learning (ML) algorithms, specifically LASSO regression and random forest (RF), to identify risk factors of disease, such as the rs28362336 genotype in HCG27. By incorporating multiomic evidence and ChIP validation, we aimed to elucidate the “functional SNP–transcription factor binding–target gene regulation” axis [20]. Our findings not only clarify the cis-regulatory mechanisms through which HCG27 modulates T-cell functions but also provide genetic markers that may facilitate the clinical stratification and personalized management of MG patients.

## Materials and methods

### Study subjects

**Fig 1:**
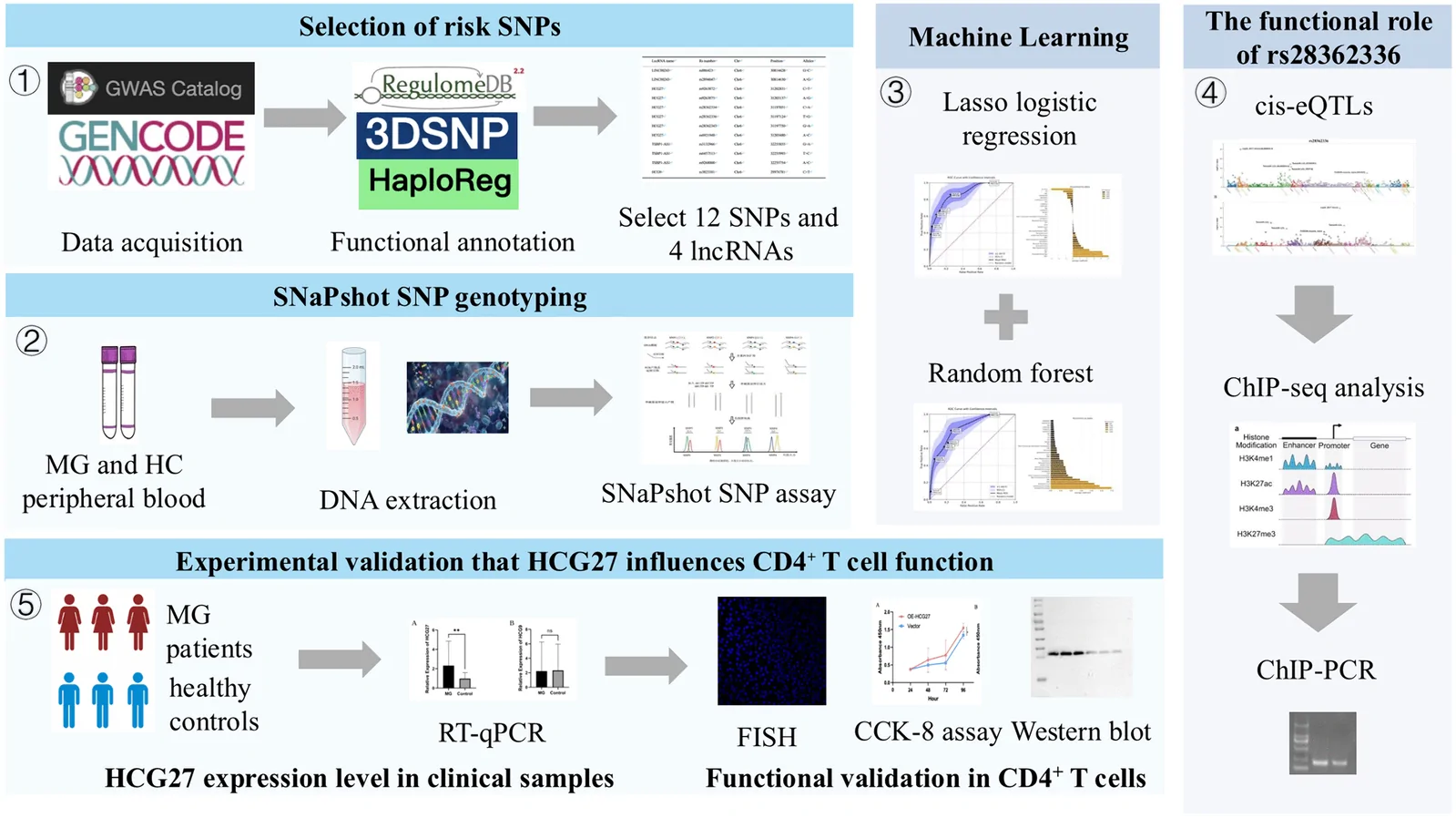
Overall research design and flowchart of the study.

An age-, sex-, and ethnicity-matched case–control study was designed to assess the association between candidate lncRNA-related SNPs and MG susceptibility (Fig 1). A total of 161 patients with MG were recruited at the Second Affiliated Hospital of Harbin Medical University between June 2024 and February 2025 and were included for genotyping. During the same period, 161 healthy controls (HCs) who underwent routine physical examinations at the same hospital were also recruited. Among the patients with MG, 131 were AChR-Ab-positive, while the antibody status of the remaining 30 patients was unknown. HCs had no personal or family history of autoimmune diseases or neuromuscular disorders. In this study, the diagnosis of MG was based on standard clinical criteria of characteristic fatigable weakness and electrophysiological and/or pharmacological abnormalities [21]. The exclusion criteria for the case group included serious cardiopulmonary diseases, severe infections, pregnancy, perinatal infections, malignancies other than thymoma, and other autoimmune diseases. Finally, 322 individuals were genotyped, including 161 MG patients and 161 HCs (S1 Table).

The studies involving human participants were approved by the Ethics Committee of the Second Affiliated Hospital of Harbin Medical University (YJSKY2024−293) and performed in accordance with the latest version of the Helsinki Declaration. The patients/participants provided their written informed consent to participate in this study.

### Data acquisition

We downloaded the dataset GCST90432156 from the GWAS Catalog (https://www.ebi.ac.uk/gwas/) released in November 2024 [22]. This dataset included 5,708 patients with MG and 432,028 controls. Among the patients with MG, 3,115 were positive for acetylcholine receptor antibodies (AChR-Ab), while 73 were not positive for AChR-Ab. These 73 patients included MuSK-Ab-positive patients, seronegative patients, and LRP4-Ab-positive patients. Antibody status information was unavailable for the remaining 2,520 patients. The long noncoding RNA gene annotation file (GTF format) was downloaded from the GENCODE database (https://www.gencodegenes.org/), which contains annotation information for lncRNAs [23]. The two datasets were downloaded on 01/12/2024. H3K4me1 (ENCFF149FZG, ENCFF785YET, and ENCFF088KUT) and H3K27ac (ENCFF779QTH, ENCFF218QBO, and ENCFF042LSM) datasets were downloaded from the ENCODE database.

### Selection of risk SNPs

To identify potential lncRNAs in which the above SNPs may be located, based on the GENCODE database, we downloaded all lncRNA data, which contain the positions of each lncRNA on the related chromosomes. The genomic positions of the candidate SNPs were compared with those of the lncRNA positions and the SNPs located within the lncRNA regions were retained for further analysis.

Initially, SNPs from the GCST90432156 dataset located on the X and Y chromosomes were excluded. Initial screening was performed based on the *P* values provided in the dataset, and SNPs with *P* < 1 × 10^-3^ were retained [24]. The genomic coordinates of the SNPs from GCST90432156 were matched with the chromosomal coordinates of the lncRNAs from GENCODE. Only SNPs overlapping with lncRNA regions were selected. Minor allele frequency (MAF) was calculated using the effect allele frequency provided in GCST90432156. SNPs with an MAF ≥ 0.05 were retained.

### SNP functional annotation

Regulatory evidence from RegulomeDB v2.2 (https://regulomedb.org/regulome-search/) was first integrated to evaluate the potential effects of SNPs on transcription factor binding, gene expression regulation, and eQTL signals [25]. According to the RegulomeDB scoring system, where a score of 1 indicates the highest regulatory potential, variants with scores from 1a to 1f were retained. We used 3DSNP v2.0 (https://omic.tech/3dsnpv2/) to assess the regulatory roles of SNPs from the perspective of three-dimensional genome organization, including annotations for enhancers, transcription factor-binding sites, and sequence motifs [26]. SNPs with scores >80 were selected. Finally, we utilized the annotation information provided by the HaploReg 4.2 (https://pubs.broadinstitute.org/mammals/haploreg/haploreg.php) as the final step for functional annotation [27]. Databases used for functional annotation were accessed on 20/12/2024.

### DNA extraction and genotyping

Genomic DNA was extracted using the Blood Genomic DNA Maxi Kit (GeneBetter Biotech, Beijing, China) and stored at –20 °C. Twelve SNP loci were genotyped using a SNaPshot assay. PCR amplification of SNP-flanking fragments was performed with Takara Hot-Start Taq (TransGen Biotech, Beijing, China). After purification using shrimp alkaline phosphatase and exonuclease I (Epicentre, Madison, America), products were subjected to primer extension using the SNaPshot Multiplex Kit (ABI, Beijing, China). Extension products were analyzed on an ABI 3130xl capillary electrophoresis instrument using GeneMapper 4.1 software (Applied Biosystems, Foster City, CA, USA). Genotyping was performed in a blinded manner without knowledge of case or control status. Additionally, 5% of the samples from both the case and control groups were randomly selected and genotyped independently by different researchers, with a repeatability rate of 100%. All primer sequences are provided in S2 Table.

### Machine learning–based identification of risk SNPs

To identify key risk SNPs, we constructed classification models using LASSO regression and RF [28,29]. Clinical data from 161 MG patients were retrospectively collected and divided into two groups: the non-exacerbation group (n = 140) and the exacerbation group (n = 21, including patients with a history of myasthenic crisis). Disease exacerbation was defined by meeting any of the following criteria: (1) a quantitative myasthenia gravis (QMG) score of ≥ 8 points and a minimum increase score of ≥ 5 points from the previous visit. Ocular findings must not account for more than 5 points on the QMG score. (2) Progressive clinical deterioration due to weakness of bulbo-pharyngeal or limb muscles or reduced respiratory function impacting activities of daily living. (3) The progression of symptoms was no longer than 30 days [30].

A total of 34 input features were included in the ML (S3 Table), and data were accessed for research purposes from 01/05/2025–15/05/2025. Model training was implemented in Python 3.10 using the scikit-learn library. To reduce the bias introduced by random data splitting, all the models were trained repeatedly 5,000 times using different random seeds (num_runs = 5000). In each run, 80% of the samples were used as the training set and 20% as the test set. For the LASSO regression, the liblinear solver with L1 regularization (C = 1.0) was applied. The frequency of nonzero coefficients and the mean coefficient value across repeated runs were calculated for each feature to reflect its stability and direction of association. For the RF, the parameters were set as n_estimators = 10 and max_depth = 3. The feature importance scores were recorded in each run, and their mean values and occurrence frequencies across iterations were calculated to assess the overall contribution of each feature in the nonlinear model. Model performance was evaluated using the area under the receiver operating characteristic curve (ROC-AUC). Furthermore, to prioritize the detection of high-risk patients and minimize false-negative predictions, we actively tuned the decision thresholds (e.g., 0.1, 0.3, and 0.8).

### Functional expression quantitative trait locus (eQTL) analysis

To analyze the regulatory functions of the tested variants, we employed the FIVEx browser (https://fivex.sph.umich.edu/) to analyze the cis-eQTLs associated with these variants and determine whether they are tissue-specific [31]. Single-tissue eQTLs were confirmed using the Genotype-Tissue Expression portal (https://www.gtexportal.org/home/) [32].

### Chromatin immunoprecipitation

Chromatin immunoprecipitation (ChIP) assays were performed using a commercial ChIP kit (Thermo Fisher Scientific, Waltham, MA, USA) according to the manufacturer’s instructions. Jurkat cells were cross-linked with 1% formaldehyde for 10 min at room temperature, and the reaction was quenched with 0.125 M glycine. Chromatin was enzymatically digested with micrococcal nuclease to obtain DNA fragments of approximately 200–1000 bp. The sheared chromatin was incubated with antibodies against RNA polymerase II (Pol II) or corresponding IgG as a negative control. Immunoprecipitation was carried out at 4 °C overnight, followed by incubation with agarose beads. After extensive washing, the chromatin-protein complexes were eluted, and cross-links were reversed in the presence of proteinase K and NaCl. The immunoprecipitated DNA was purified using spin columns and subsequently analyzed by quantitative PCR.

### Total RNA extraction and real-time PCR

A total of 35 patients with MG and 34 healthy controls (HCs) were recruited for peripheral blood RNA extraction (S4 Table). All 35 patients had newly diagnosed, new-onset MG and were treatment-naive, with no prior use of immunosuppressive agents. The 34 healthy controls were selected from individuals undergoing routine health examinations during the same period and were matched to the patients by age and sex. Peripheral blood mononuclear cells (PBMCs) were isolated using lymphocyte separation medium and stored at –80 °C. Total RNA was extracted using TRIzol reagent (SEVEN, Beijing, China) following the manufacturer’s instructions. cDNA was synthesized using SevenCleverTM First Strand cDNA Synthesis Kit (SEVEN, Beijing, China). Quantitative real-time PCR (qRT-PCR) was performed using the 2 × SYBR Green qPCR MasterMixⅡ(SEVEN, Beijing, China) to assess HCG27 and HCG9 expression. GAPDH was used as an internal control for lncRNAs (S5 Table). The melting curve of each sample is single-peaked, indicating specific PCR amplification. The results were quantified as the fold change between the expression of GAPDH and that of the target gene using the formula 2^−ΔΔCT^.

### Fluorescence in situ hybridization (FISH)

FISH assays were carried out using a Fluorescence in Situ Hybridization Kit (RiboBio Biotechnology, Guangzhou, China). The suspended cells fixed on slides were washed with PBS and fixed with 4% formaldehyde at room temperature for 10 min. Then, the cells were permeabilised with PBS containing 0.5% Triton X-100 (Saiguo Biotechnology, Guangzhou, China) on ice for 5 min. Hybridization was performed by incubating the cells with a Cy3-labeled probe at 37 °C overnight. After the probes were removed, the cells were stained with 4’,6-diamidino-2-phenylindole (DAPI) at room temperature for 5 min. Images were acquired with a confocal microscope (LSM980, Zeiss) to analyze the localization of HCG27.

### Cell culture and transfection

Jurkat cells (a CD4 + T-cell line) were purchased from the BeNa Culture Collection (Henan, China). Jurkat cells were maintained in RPMI 1640 medium (Gibco, USA) supplemented with 10% fetal bovine serum (FBS, Excell Bio, Suzhou, China) and 1% penicillin–streptomycin (Seven, Beijing, China). All the cells were incubated at 37 °C in a humidified atmosphere with 5% CO_2_. Cells at 6–10 passages were used for subsequent experiments.

Jurkat cells in the logarithmic growth phase were centrifuged (1000 rpm, 5 min) and seeded into 6-well plates at a density of 1 × 10^5^ cells/well. PcDNA3.1-HCG27 and si-HCG27 were constructed by GenePharma (Shanghai, China). Cells were transfected with PcDNA3.1-HCG27, si-HCG27, or corresponding negative controls according to the experimental design. Plasmid overexpression and siRNA delivery were performed with GP-transfect-Mate Transfection Reagent and siRNA-mate Plus transfection kit (GenePharma, Shanghai, China), respectively, both strictly following the manufacturer’s protocols. Transfected cells were incubated for 48 h (for total RNA extraction for qRT‒PCR) to evaluate transfection efficiency.

### Cell proliferation

Cell proliferation was evaluated using the Cell Counting Kit-8 (CCK-8; Seven, Beijing, China). Briefly, transfected Jurkat cells were seeded into 96-well plates at a density of 2.5 × 10^3^ cells per well in 100 μL of complete medium and incubated at 37 °C in a humidified atmosphere containing 5% CO_2_. At 24, 48, 72, and 96 h after transfection, 10 μL CCK-8 reagent was added to each well, followed by incubation for 2 h at 37 °C. The absorbance was then measured at 450 nm using a microplate reader (BioTek, USA).

### Western blot analysis

The cells were collected and lysed in radioimmunoprecipitation assay buffer (P0013B, Beyotime) supplemented with 1% protease and phosphatase inhibitors (Roche, Basel, Switzerland). The total protein concentrations were measured using a Bicinchoninic Acid Protein Assay Kit (Seven, Beijing, China). Sample proteins were separated by SDS‒polyacrylamide gel electrophoresis and transferred onto polyvinylidene difluoride membranes (Roche, Basel, Switzerland). Then, the membranes were blocked in protein-free rapid blocking buffer (YY101, Epizyme Biomedical) for 0.5 h at room temperature overnight at 4 °C before being incubated with various primary antibodies. The following primary antibodies were used: rabbit anti-Bcl-2 (12789–1-AP, 1:2000, Proteintech), anti-BAX (WL01637, 1:1000, Proteintech), and anti-beta-actin (AF7018, 1:10000, Affinity). The membranes were then incubated with the corresponding secondary antibodies (Immunoway, 1:10000, China) for 1 h the following day. After the membranes were washed with TBST, protein expressions were visualized with enhanced chemiluminescence reagent (Meilunbio, China).

### Data analysis

Statistical analysis and graphing were performed with GraphPad Prism 9.0. Genotypes and allele frequencies were obtained through direct counting. Differences between groups were compared using the chi-square test or Fisher’s exact probability test, as appropriate. The relative risk associated with alleles and genotypes was estimated as an odds ratio (OR) with a 95% confidence interval (CI). For the qRT‒PCR, statistical analysis was performed using the 2^−ΔΔCT^ values. Student’s t test was used for comparisons of the differences between two groups. Correlation analysis was performed using Pearson correlation. All the data are presented as the mean ± standard deviation (SD). *P* values < 0.05 were considered statistically significant.

## Results

### Screening and identification of MG-associated lncRNA SNPs

First, approximately 7.12 million MG-associated SNPs were obtained from GCST90432156, and 20,129 lncRNAs were downloaded from the GENCODE database. Furthermore, for lncRNAs and MG-associated SNPs on the same chromosome, we matched them with the SNP position and lncRNA sequencing position range to find the candidate SNPs that might be located within related lncRNAs. Then, we obtained 685 SNPs that might be located within 175 lncRNAs. After minor allele frequency (MAF) filtering, 603 SNPs within 152 lncRNAs were retained.

To further prioritize the functional variants among these MG-associated candidates, SNPs with RegulomeDB scores of 1a–1f were selected, yielding 475 SNPs. Then, based on annotations from the 3DSNP 2.0, SNPs with scores >80 were further selected, resulting in 93 SNPs. The intersection of the SNPs from the two databases yielded 65 SNPs. Finally, by integrating regulatory annotation information from the HaploReg v4.2, 12 SNPs located in four lncRNAs were identified as the most promising functional variants potentially involved in the pathogenesis of MG (S6 Table). These SNPs are located within the genomic regions of LINC00243, HCG27, TSBP1-AS1, and HCG9 (S7 Table).

### Analysis of the distribution of SNPs on chromosomes

**Fig 2:**
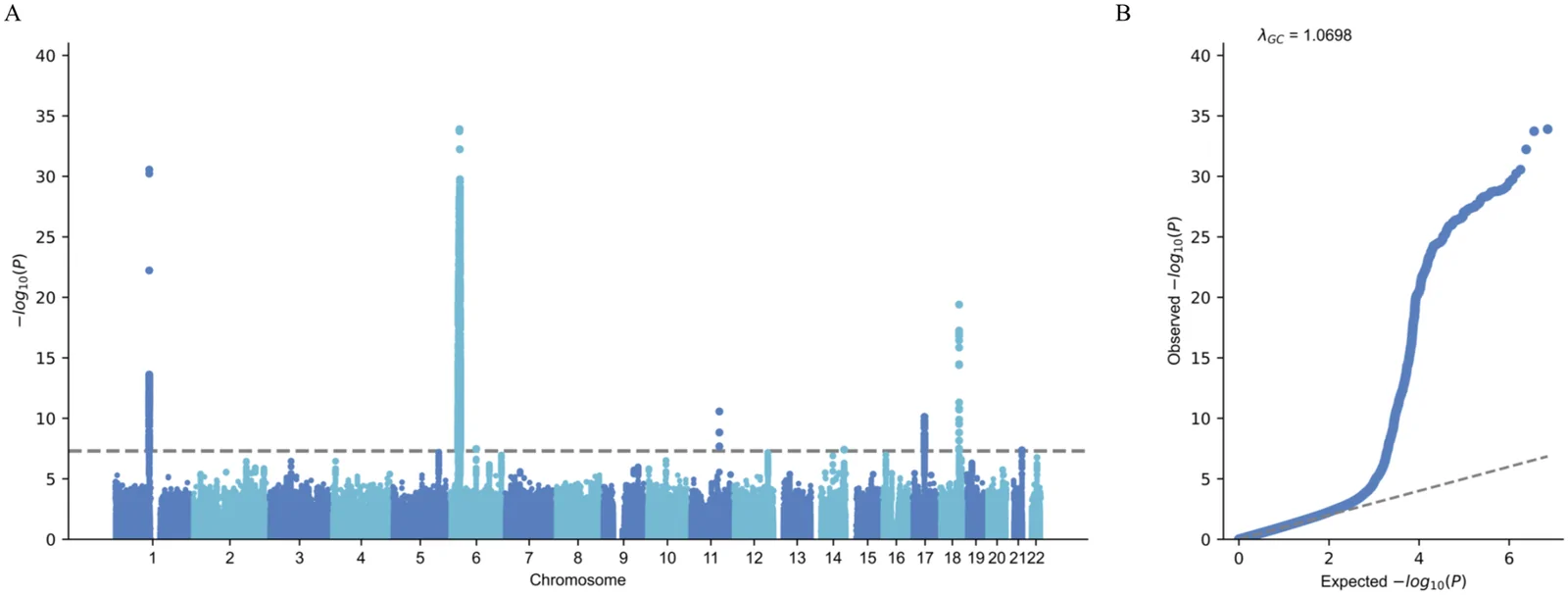
Manhattan plot (A) and Q–Q plot (B) of the GCST90432156 dataset. A The x-axis represents the chromosome, and the y-axis represents the − log10 (*P*) value. The dots represent the − log10 (*P*) values of each SNP in the GCST90432156 dataset. B The x-axis represents the expected −log10 (*P*) values, and the y-axis represents the observed −log10 (*P*) values.

Based on the GCST90432156 dataset, the genome-wide distribution of SNPs associated with MG was analyzed. The Manhattan plot revealed highly significant SNPs on chromosomes 1, 6, 11, 17, and 18, with the strongest association signal observed on chromosome 6 (Fig 2A). This finding is consistent with previous studies [14]. In the QQ plot, a certain deviation between the expected and observed P values can be observed (Fig 2B). However, the overall distribution trend remains generally consistent. The genomic inflation factor was λ = 1.0698 (λ < 1.1), indicating that the overall SNP statistical bias was minimal.

### Distribution of genotypes and alleles of lncRNA SNPs and the risk of MG

All the SNP loci in the control group were in accordance with Hardy‒Weinberg equilibrium (HWE, *P* > 0.05). In a case‒control study comprising 161 MG patients and 161 HCs, the overall analysis revealed no significant differences in the genotype or allele frequencies of the 12 candidate SNPs between the two groups (S8 Table, S9 Table). The basic information of these 12 SNPs is shown in S7 Table. Furthermore, no significant differences were observed in the genotype or allele frequencies of these 12 SNPs between early-onset myasthenia gravis (EOMG) patients and late-onset myasthenia gravis (LOMG) patients (S10 Table).

However, as shown in Table 1, stratified analysis based on clinical subtypes revealed significant associations: three SNP loci within lncRNA HCG27 were significantly associated with the risk of gMG. Compared with gMG, the frequencies of the rs9263872 TT genotype (OR = 0.320; 95% CI = 0.111–0.921; *P* = 0.03), rs9263875 GG genotype (OR = 0.180; 95% CI = 0.049–0.663; *P* = 0.006), and rs28362336 GG genotype (OR = 0.284; 95% CI = 0.106–0.877; *P* = 0.023) were significantly different in oMG patients. Notably, for rs9263875, the G allele was significantly less frequent in patients with oMG than in those with gMG (OR = 0.571; 95% CI = 0.357–0.911; *P* = 0.021). With respect to lncRNA HCG9, the rs3823381 TT genotype (OR = 2.846; 95% CI = 1.052–7.699; *P* = 0.036) and the T allele (OR = 1.600; 95% CI = 0.999–2.500; *P* = 0.045) were significantly associated with oMG. After FDR correction, rs28362336 (FDR-adjusted *P* = 0.044), rs9263872 (FDR-adjusted *P* = 0.044), and rs9263875 (FDR-adjusted *P* = 0.044) remained statistically significant (S11 Table). Additionally, a significant difference in the distribution of the HCG9 rs3823381 TT genotype (OR = 4.200; 95% CI = 1.369–11.280; *P* = 0.009) and the T allele (OR = 1.700; 95% CI = 1.078–2.682; *P* = 0.022) was associated with female MG patients (S12 Table). After Benjamini–Hochberg FDR correction, none of the genotype- or allele-level associations with sex remained statistically significant.

**Table 1:** Distribution of SNP genotype and allele frequencies in ocular MG and generalized MG.

SNP	Genotype/Allele	Subgroups		χ^2^	*P*	OR (95% CI)
		oMG	gMG			
rs886423	GG	53(93.0)	98(94.2)			
	GC	4(7.0)	6(5.8)	-	0.744	1.233(0.379,4.902)
	G	110(96.5)	202(97.1)			
	C	4(3.5)	6(2.9)	-	0.747	1.224(0.383,4.661)
rs2894047	AA	11(19.3)	15(14.4)			
	AG	24(42.1)	60(57.7)	1.727	0.189	0.545(0.219,1.356)
	GG	22(38.6)	29(27.9)	0.005	0.945	1.034(0.398,2.689)
	A	46(40.4)	90(43.3)			
	G	68(59.6)	118(56.7)	0.256	0.612	1.127(0.704,1.780)
rs9263872	CC	17(29.8)	29(27.9)			
	CT	34(59.6)	43(41.3)	0.614	0.433	1.349(0.638,2.852)
	TT	6(10.5)	32(30.8)	4.689	0.030	0.320(0.111,0.921)
	C	68(59.6)	101(48.6)			
	T	46(40.4)	107(51.4)	3.633	0.057	0.639(0.405,1.018)
rs9263875	AA	24(42.1)	36(34.6)			
	GA	30(52.6)	43(41.3)	0.016	0.898	1.047(0.522,2.099)
	GG	3(5.3)	25(24.0)	-	0.006	0.180(0.049,0.663)
	A	78(68.4)	115(55.3)			
	G	36(31.6)	93(44.7)	5.289	0.021	0.571(0.357,0.911)
rs28362334	CC	48(84.2)	90(86.5)			
	CA	9(15.8)	14(13.5)	0.163	0.686	1.205(0.508,3.082)
	C	105(92.1)	194(93.3)			
	A	9(7.9)	14(6.7)	0.15	0.698	1.188(0.472,2.809)
rs28362336	TT	17(29.8)	29(27.9)			
	TG	35(61.4)	45(43.3)	0.556	0.456	1.327(0.612,2.828)
	GG	5(8.8)	30(28.8)	5.164	0.023	0.284(0.106,0.877)
	T	69(60.5)	103(49.5)			
	G	45(39.5)	105(50.5)	3.585	0.058	0.640(0.405,1.022)
rs28362343	GG	48(84.2)	90(86.5)			
	GA	9(15.8)	14(13.5)	0.163	0.686	1.205(0.508,3.082)
	G	105(92.1)	194(93.3)			
	A	9(7.9)	14(6.7)	0.15	0.698	1.188(0.472,2.809)
rs6921948	AA	21(36.8)	44(42.3)			
	AC	29(50.9)	45(43.3)	0.712	0.399	1.350(0.671,2.715)
	CC	7(12.3)	15(14.4)	0.002	0.966	0.978(0.347,2.758)
	A	71(62.3)	133(63.9)			
	C	43(37.7)	75(36.1)	0.088	0.767	1.074(0.667,1.701)
rs3132966	GG	38(66.7)	64(61.5)			
	GA	18(31.6)	38(36.5)	0.413	0.521	0.798(0.400,1.590)
	AA	1(1.8)	2(1.9)	-	1.000	0.842(0.074,9.602)
	G	94(82.5)	166(79.8)			
	A	20(17.5)	42(20.2)	0.332	0.564	0.841(0.465,1.530)
rs6457513	TT	37(64.9)	64(61.5)			
	TC	19(33.3)	38(36.5)	0.173	0.677	0.865(0.437,1.713)
	CC	1(1.8)	2(1.9)	-	1.000	0.865(0.076,9.867)
	T	93(81.6)	166(79.8)			
	C	21(18.4)	42(20.2)	0.147	0.702	0.893(0.504,1.606)
rs9268000	AA	24(42.1)	41(39.4)			
	AC	29(50.9)	54(51.9)	0.062	0.803	0.917(0.467,1.804)
	CC	4(7.0)	9(8.7)	-	0.761	0.759(0.211,2.734)
	A	77(67.5)	136(65.4)			
	C	37(32.5)	72(34.6)	0.153	0.695	0.908(0.564,1.465)
rs3823381	CC	13(22.8)	37(35.6)			
	CT	31(54.4)	54(51.9)	1.571	0.210	1.634(0.756,3.532)
	TT	13(22.8)	13(12.5)	4.378	0.036	2.846(1.052,7.699)
	C	57(50.0)	128(61.5)			
	T	57(50.0)	80(38.5)	4.011	0.045	1.600(0.999,2.500)

OR, odds ratio; 95% CI, 95% confidence interval. The reference group is the gMG group.

### Identification of key risk feature rs28362336 for MG based on machine learning algorithms

To explore the key factors associated with MG, we constructed LASSO regression and RF models. Baseline characteristics were also compared between the two groups. The proportion of patients receiving baseline immunosuppressive therapy differed significantly between the two groups (P = 0.016; S13 Table).

**Fig 3:**
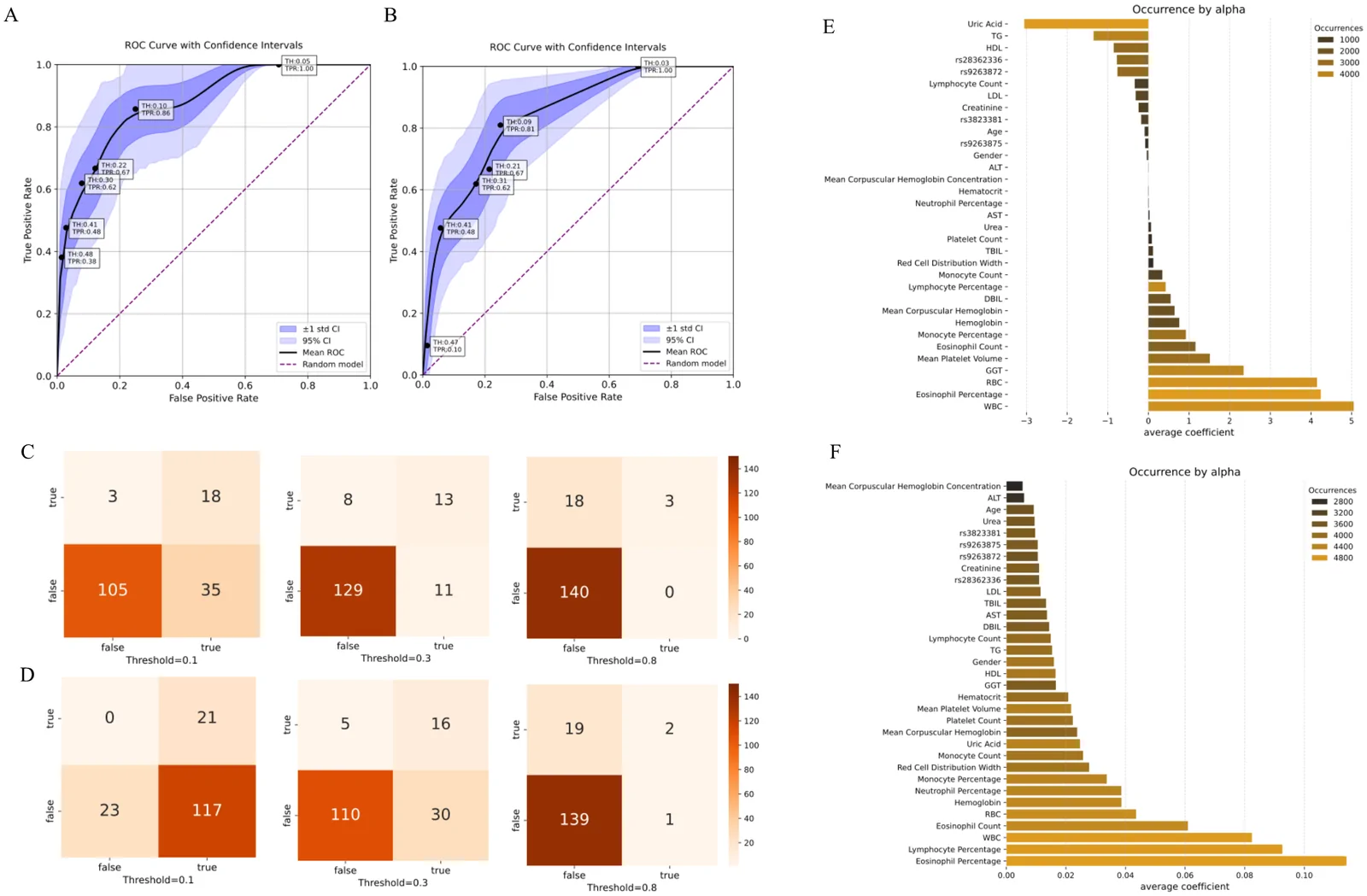
Machine learning models identify rs28362336 as a key factor of MG. A–B Prediction results independent of threshold for the LASSO regression and random forest. The black line represents the mean ROC curve. The shaded dark blue area represents ± 1 standard deviation (SD), and the light blue area represents ± 2 SDs. C–D Comparison of model classification performance under different threshold settings. Prediction on the x-axis and ground truth on the y-axis. E–F Feature importance and stability analysis.

The LASSO regression exhibited a higher AUC (0.877) than the RF (0.858) (Fig 3A and 3B). To identify patients at high risk of disease, three decision thresholds (0.1, 0.3, and 0.8) were applied to construct confusion matrices. The LASSO model was evaluated at thresholds of 0.8, 0.3, and 0.1. As the threshold decreased from 0.8 to 0.1, false negatives decreased from 18 to 3, while false positives increased from 0 to 35 (Fig 3C). The RF model showed a similar trend but produced more false-positive predictions at lower thresholds (Fig 3D). These results suggest that the LASSO model with a lower threshold may be more suitable for screening and reducing missed high-risk patients.

Feature importance analysis using LASSO regression and RF (Fig 3E and 3F). Notably, the HCG27 SNP rs28362336 emerged as a critical marker with high contribution and exceptional stability across both models. In the LASSO, rs28362336 was consistently selected through repeated iterations with a stable regression coefficient, while the RF confirmed its high average feature importance with minimal fluctuation across multiple iterations. Taken together, the consistent retention of rs28362336 in the LASSO model, together with its contribution in the RF analysis, supports its prioritization as a candidate genetic predictor for subsequent functional validation.

### Results of eQTL analysis

**Fig 4:**
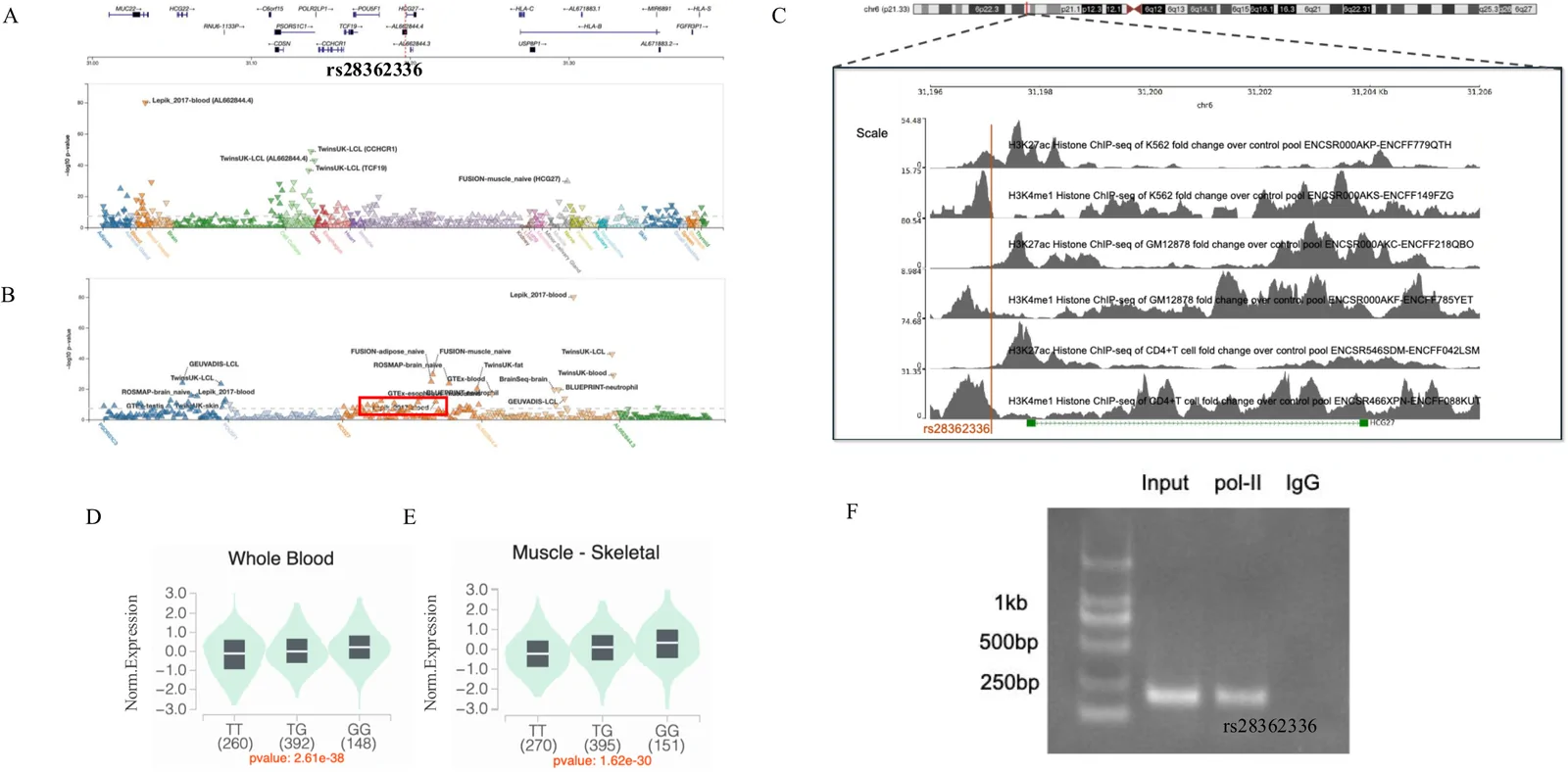
Regulatory and functional effects of the MG-associated SNP rs28362336 on HCG27 expression. A A variant-centric view of eQTL *P*-values for rs28362336. This variant is a strong cis-eQTL regulating multiple genes. B The same view shows signals in different systems, with a strong signal appearing for HCG27 in the muscle. A highly significant association with HCG27 expression was also observed in peripheral blood. Data were generated from the FIVEx browser (https://fivex.sph.umich.edu/). HCG27, HLA complex group 27. C Enrichment of H3K4me1 and H3K27ac histone modifications in K562, GM12878, and CD4 + T cells based on ChIP-seq data. D–E eQTL analysis demonstrating the significant regulatory effects of different genotypes (TT, TG, GG) on HCG27 expression levels in whole blood (D) and muscle tissue (E). F ChIP analysis of Pol II enrichment in Jurkat cells.

We examined the cis-eQTLs associated with the tested genetic variants to elucidate their potential tissue-specific regulatory effects. According to the FIVEx browser, rs28362336 is a strong cis-eQTL associated with the expression of multiple genes. Among these associations, the strongest was observed for HCG27 expression in muscle tissue (−log10 *P* value = 29.62, effect size = 0.33, positive effect). A highly significant association with HCG27 expression was also observed in peripheral blood. Association signals from multiple independent datasets were distributed around the rs28362336 locus. The signal cluster overlapped with the genomic region containing HCG27, suggesting that the association signal may represent a true functional locus in this region (Fig 4A and 4B). Notably, strong signals were also observed in datasets derived from immune-related tissues and blood cells, which are biologically relevant to the pathogenesis of MG.

### rs28362336 regulated the expression of HCG27 as an enhancer

Bioinformatics analysis using ENCODE (https://www.encodeproject.org/) ChIP-seq data revealed that the rs28362336 locus upstream of HCG27 is enriched for enhancer-associated histone marks, including H3K4me1 and H3K27ac, suggesting regulatory enhancer activity (Fig 4C). Subsequent ChIP experiments confirmed significant binding of RNA polymerase II (Pol II) at the rs28362336 locus, whereas no enrichment was observed for the IgG control (Fig 4F). These findings indicate that rs28362336 resides in a transcriptionally active regulatory region capable of recruiting the transcriptional machinery. Consistently, eQTL analysis of whole blood revealed that rs28362336 is significantly associated with HCG27 expression, with the G allele correlated with increased expression and the GG genotype showing the highest expression levels (Fig 4D and 4E). The ENCODE SCREEN database (https://screen.encodeproject.org/) additionally revealed that rs28362336 overlaps with an enhancer-like candidate cis-regulatory element, which is supported by strong DNase hypersensitivity and CTCF signals (S1 Fig).

Taken together, these results indicate that rs28362336 regulates HCG27 expression in an allele-dependent manner and is likely a functional regulatory variant.

### Subcellular localization of HCG27 in CD4 + T cells and its function in promoting CD4 + T cell proliferation

**Fig 5:**
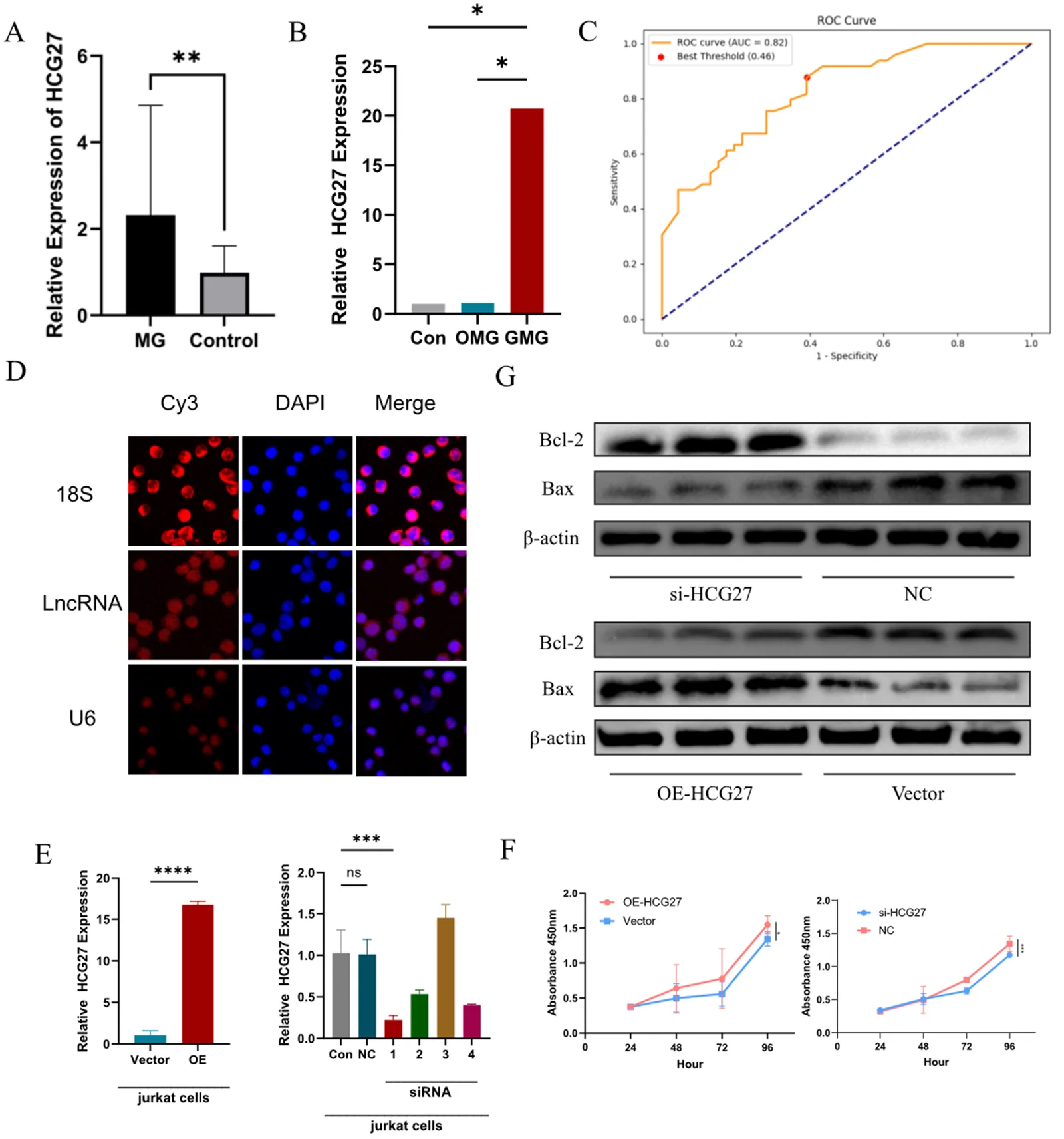
HCG27 promoted CD4 + T cell proliferation and inhibited CD4 + T cell apoptosis. A The expression of HCG27 was detected in MG patients and HCs by qRT‒PCR. B The expression of HCG27 was detected in clinical samples. C ROC curve analysis of HCG27 levels. D Subcellular localization of HCG27 in Jurkat cells. E The efficiency of HCG27 knockdown and overexpression was measured by qRT‒PCR. F The proliferation of Jurkat cells was analyzed using CCK-8 assays by transfecting negative controls, OE-HCG27, or si-HCG27 into Jurkat cells. G Western blot analysis of Bcl-2 and Bax in the OE-HCG27, vector, si-HCG27 and NC group. β-actin served as the internal loading control. The data are presented as the mean ± SD (*P < 0.05, ** *P* < 0.01, *** *P* < 0.001, **** *P* < 0.0001).

The qRT‒PCR results demonstrated that the expression level of HCG27 was significantly greater in the PBMCs of MG patients than in those of HCs (*P* < 0.01) (Fig 5A). Genotype‒phenotype association analysis revealed that compared with oMG patients and HCs, gMG patients carrying the risk genotype GG at rs28362336 exhibited significantly higher HCG27 expression levels in their PBMCs (*P* < 0.05) (Fig 5B). These findings suggest that rs28362336 may upregulate HCG27 expression via its enhancer activity, thereby promoting progression of the disease to the generalized form.

We performed ROC curve analysis to evaluate the diagnostic performance of HCG27 based on expression level, and the results revealed that the lncRNA accurately distinguished MG patients from HCs (AUC = 0.873; Fig 5C). We first evaluated the subcellular localization of HCG27 in Jurkat cells. FISH was performed to determine the localization of HCG27 in Jurkat cells, and the results indicated that HCG27 predominantly localized to the nucleus and cytoplasm (Fig 5D). CCK-8 assays revealed that following transfection with the pcDNA3.1-HCG27 overexpression plasmid, the proliferation capacity of Jurkat cells significantly increased over time (*P* < 0.05). Conversely, the proliferation of Jurkat cells was significantly inhibited after knocking down HCG27 expression via transfection with specific siRNAs (Fig 5E and 5F). In summary, HCG27 promotes the proliferation of CD4 + T cells, the key immune cells in MG, suggesting that its high expression may participate in the pathological process of MG patients through the expansion of the pathogenic T cell population. Western blot analysis demonstrated that HCG27 overexpression significantly upregulated the expression of the anti-apoptotic protein Bcl-2 while downregulating the expression of the pro-apoptotic protein Bax (Fig 5G). Conversely, HCG27 expression knockdown induced the opposite pattern of protein expression. These findings are consistent with the results of the CCK-8 assay, collectively providing evidence that HCG27 functions to promote proliferation and inhibit apoptosis in cells.

## Discussion

**Fig 6:**
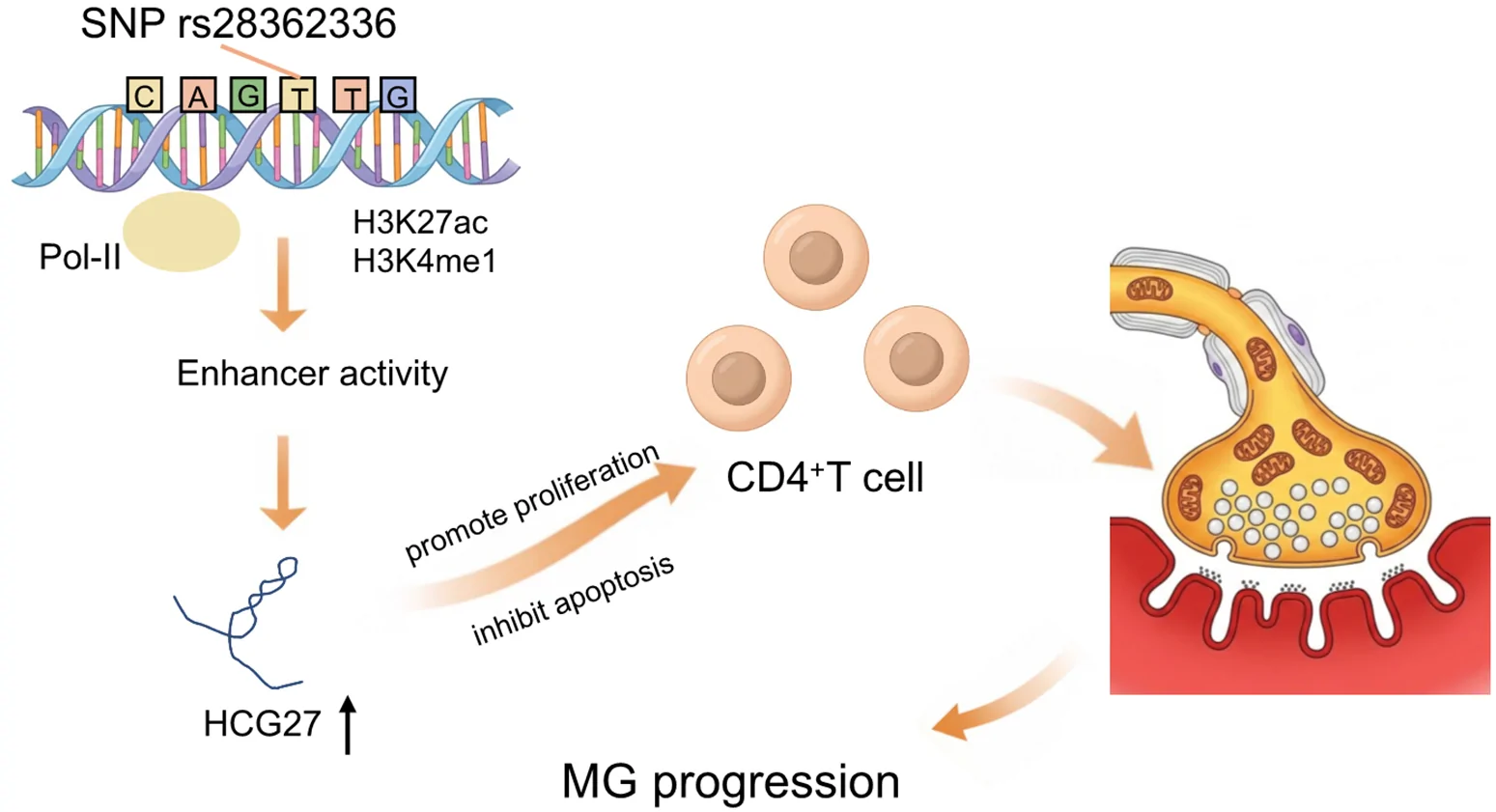
Mechanism diagram.

MG is a complex autoimmune disease in which genetic susceptibility plays a crucial role in disease onset and progression [1]. Gaining an in-depth understanding of the intricate relationship between genetic discoveries and disease holds paramount importance in effectively translating GWAS findings into clinical applications [12,33]. We screened candidate SNPs using a large-scale GWAS dataset of patients diagnosed with MG and performed lncRNA SNP genotyping in the peripheral blood of MG patients and HCs using a SNaPshot assay. Crucially, our findings reveal distinct genetic mechanisms underlying oMG and gMG, as evidenced by differential SNP distributions at identical loci across MG subtypes. Using LASSO regression and RF, we identified the rs28362336 GG genotype as a key factor of disease. Further analysis revealed enrichment of enhancer-associated histone modifications at this site. Experimental validation suggests that rs28362336 functions via a “functional SNP–transcription factor binding–target gene regulation” axis, providing novel insights into the divergent genetic basis of MG (Fig 6).

Extensive genetic studies have established that functional single SNPs are associated with various autoimmune diseases [34]. Notably, many GWAS-identified SNPs reside within noncoding regions, suggesting that noncoding RNAs play a pivotal role in disease pathogenesis [35]. To identify potential functional variants, we evaluated candidate SNPs using many databases, integrating functional annotations such as transcription factor binding affinity, gene expression regulation, histone modifications, and eQTL evidence. Consequently, 12 candidate SNPs were prioritized for further investigation. Subsequent genotyping and allelic frequency analysis demonstrated that rs3823381 in lncRNA HCG9 showed a nominal association with sex, although this association did not remain statistically significant after FDR correction. Furthermore, several SNPs exhibited subtype-specific associations: rs9263872, rs9263875, and rs28362336 in lncRNA HCG27 were significantly associated with gMG. These findings suggest that genetic variations may contribute to the divergent molecular regulatory mechanisms underlying MG subtypes.

Advances in GWAS technology have led to the identification of multiple genetic variants within the MHC region that are significantly associated with MG risk [4]. Specifically, the HLA gene cluster at chromosome 6p21.3 is a critical susceptibility region. This region contains not only protein-coding genes but also an abundance of noncoding RNAs. Among these regions, HCG27 is a lncRNA located within the 6p21 (HLA) region. Notably, linkage disequilibrium analysis showed that rs28362336 was not in strong LD with previously reported HLA tag SNPs [26]. While prior studies have linked HCG27 to autoimmune diseases such as pemphigus and systemic lupus erythematosus, its specific role and underlying mechanisms in MG remain unexplored [16].

At the expression level, HCG27 expression was significantly upregulated in the PBMCs of MG patients compared with those of HCs, suggesting a potential pathogenic role. Furthermore, among individuals carrying the rs28362336 GG genotype, HCG27 expression differed significantly between oMG and gMG patients. Given that this locus is enriched in enhancer-associated histone modifications and Pol II binding signals, we hypothesize that rs28362336 resides within a transcriptionally active functional enhancer. The HCG27 SNP rs28362336 has a strong predicted regulatory function (RegulomeDB score = 1b, indicating TF binding + any TF binding motif + DNase Footprint + DNase peak + eQTL) [25]. As an enhancer SNP, it may recruit transcription complexes to upregulate HCG27 expression, thereby contributing to the genetic regulation of different MG clinical subtypes [36]. This cis-regulatory mechanism explains the high expression of HCG27 in gMG patients with the GG genotype and highlights the divergent pathological mechanisms between MG subtypes.

Although HCG27 has been documented in other contexts, its role in MG remains poorly understood. Since MG is a T cell-dependent, antibody-mediated disease, T cell activation and proliferation are central to its pathogenesis [37]. Using Jurkat cells as an in vitro model, we evaluated the effects of HCG27 on CD4 + T cell apoptosis and proliferation. Our results show that HCG27 promotes T cell proliferation and inhibits apoptosis, indicating that it contributes to MG progression by modulating T cell functions. Notably, prior studies reported abnormal HCG27 expression in patients with SLE [16]. Coexpression network analysis in SLE revealed a close link between HCG27 and CD40LG, a key molecule that mediates T–B cell interactions. These findings suggest that HCG27 may operate through a similar immune regulatory axis in MG patients, further supporting the biological and pathological significance of its upregulation in our cohort.

However, given the substantial pathological heterogeneity of MG, the lack of patient stratification by disease subtype may have introduced some background noise. Although relatively rigorous internal validation was performed in the machine-learning analysis, this study lacked an independent external validation cohort. Accordingly, future studies will validate and calibrate the model in independent, multicenter cohorts with broader population representation. This will allow further assessment of the model’s stability and potential clinical utility.

## Conclusion

This study systematically investigated the role of lncRNA-related SNPs in MG. We identified rs28362336 in HCG27 as a potential functional enhancer variant associated with gMG. Bioinformatic and experimental analyses demonstrated that rs28362336 may regulate HCG27 expression through enhancer activity, and functional assays revealed that HCG27 promotes CD4 + T cell proliferation. These findings provide new insight into the genetic regulatory mechanisms underlying MG and highlight lncRNA-associated regulatory variants as key factors for disease therapeutic targeting.

## Supporting information

S1 Fig ENCODE SCREEN annotation of the rs28362336 locus upstream of HCG27. (TIFF)

S1 Table Clinical features of DNA samples. (DOCX)

S2 Table Genotyping primers. (DOCX)

S3 Table Feature name. (DOCX)

S4 Table Clinical features of RNA samples. (DOCX)

S5 Table The primers used for real‐time PCR. (DOCX)

S6 Table Twelve potentially functional SNP and their corresponding lncRNAs identified via database screening. (DOCX)

S7 Table Basic information of the included SNP loci. (DOCX)

S8 Table Distribution of genotype and allele frequencies between the MG group and healthy controls. (DOCX)

S9 Table SNaPshot genotyping results for the 12 candidate SNPs in patients with MG and healthy controls. (XLSX)

S10 Table Distribution of SNP genotype and allele frequencies in groups stratified by age. (DOCX)

S11 Table Benjamini–Hochberg false discovery rate-adjusted comparison of SNP genotype distributions between ocular and generalized myasthenia gravis. (XLSX)

S12 Table Distribution of SNP genotype and allele frequencies in groups stratified by gender. (DOCX)

S13 Table Baseline immunosuppressive therapy in patients with myasthenia gravis. (XLSX)

S1 Raw images Western blot images. (PDF)

S1 File Pol II enrichment at the rs28362336 locus. (TIF)

## Abbreviations

MG: Myasthenia gravis
HCs: Healthy controls
lncRNAs: Long noncoding RNAs
SNPs: Single-nucleotide polymorphisms
PBMCs: Peripheral blood mononuclear cells
AChR: Acetylcholine receptor
LRP4: Low-density lipoprotein receptor-related protein 4
MuSK: Muscle-specific kinase
EOMG: Early-onset myasthenia gravis
LOMG: Late-onset myasthenia gravis
oMG: Ocular myasthenia gravis
gMG: Generalized myasthenia gravis
qRT‒PCR: Quantitative real-time PCR
HWE: Hardy‒Weinberg equilibrium
OR: Odds ratio
CI: Confidence interval
FISH: Fluorescence in situ hybridization
H3K27ac: Histone H3 lysine 27 acetylation
H3K4me: Histone H3 lysine 4 monomethylation
HLA: Human leukocyte antigen
MHC: Major histocompatibility complex
QMG: Quantitative myasthenia gravis
ML: Machine learning
RF: Random forest
ROC: Receiver operating characteristic
SLE: Systemic lupus erythematosus
