# Supplementary material for: Identification of rs28362336 as a risk SNP for generalized myasthenia gravis and its impact on HCG27 regulation
Source: PLoS One. 2026 Sep 23;21(9):e0359069. doi: 10.1371/journal.pone.0359069 (PMC13600526; doi:10.1371/journal.pone.0359069)
Supplement: Supplementary file 2 [file pone.0359069.s002.docx]

S1_Table. Clinical features of DNA samples.

| Characteristic | MG  (n=161) | Control  (n=161) |
| --- | --- | --- |
| Age (y) | 60.89 ± 13.26 | 60.91 ± 11.14 |
| Gender (M/F) | 67/94 | 67/94 |
| EOMG (≤ 50 y) | 28 | - |
| LOMG (>50 y) | 133 | - |
| OMG | 59 | - |
| GMG | 102 | - |
| AChR Ab (Positive/Total) | 131/161 | - |

Among the 161 patients with MG, 131 patients had MG-related antibody test and 30

patients refused MG-related antibody test.
