# Supplementary material for: Identification of rs28362336 as a risk SNP for generalized myasthenia gravis and its impact on HCG27 regulation
Source: PLoS One. 2026 Sep 23;21(9):e0359069. doi: 10.1371/journal.pone.0359069 (PMC13600526; doi:10.1371/journal.pone.0359069)
Supplement: Supplementary file 3 [file pone.0359069.s003.docx]

S2_Table. Genotyping primers.

| LncRNA | SNP | PCR primer |  | SNaPshot primer |
| --- | --- | --- | --- | --- |
| LINCC00243 | rs886423 | F: TGGAGCAACTCCAATGTCACCT | SF: | TTTTTTTTTTTTTTTTTTTTTTTTTTTTTTTTTTTTTTTTTTTTTTTTTCTCAGACTGCCTTGAATCCAGG |
|  |  | R: CAAGATGGTTGCTCTGCCTTCA |  |  |
|  | rs2894047 | F: TGGAGCAACTCCAATGTCACCT | SR: | TTTTTTTTTTTTTTGCTCTGCCTTCAGACATCACAT |
|  |  | R: CAAGATGGTTGCTCTGCCTTCA |  |  |
| HCG27 | rs9263872 | F: ACTCACACATCACCCCTTATTTGGAA | SF: | TTTTTTTCTCACTCTAACCTTCCTTCCTGC |
|  |  | R: GAAAGAGACAGCAGGAGGGATCA |  |  |
|  | rs9263875 | F: ACTCACACATCACCCCTTATTTGGAA | SR: | TTTTTTTTTTTTTTTTTTTTTTTTTTTTTTAAGATTTTTCTTTCCTGTGGAGTATTT |
|  |  | R: GAAAGAGACAGCAGGAGGGATCA |  |  |
|  | rs28362334 | F: TCTTCAACACGTCCTCCCATC | SR: | TTTTTTTTTTTTTTTTTTTTTTTTTTTTTTTTTTTTTTTTTTTTTGGGGTCAAATGCAGAGTGGA |
|  |  | R: CCCATCAAAGTCTGCAGTGACA |  |  |
|  | rs28362336 | F: TCTTCAACACGTCCTCCCATC | SR: | TTTTTTTTTTTTTTTTTTTTTTTTTTTTTTTTTTTTTTTTTTTGCCTCGCGTCCTTTAYTTCA |
|  |  | R: CCCATCAAAGTCTGCAGTGACA |  |  |
|  | rs28362343 | F: ACGCAGCAGCAGGAAGAGTTTC | SR: | TTTTTTTTTTTTTTTTTGGTGAGTAGAGAAATGAGCCTCAG |
|  |  | R: CGGATTCGGAGACAGGTGAGTAGA |  |  |
|  | rs6921948 | F: GAGTCATTTATTTGCCTGTGTAAGCTC | SR: | TTTTTTTTTTTTTTTTTTTTTTTTTTTTTTTTTTTTTTTTTTCGYTATGTCCCGAGGACATG |
|  |  | R: GCAAAATAAACTGTACACCCTGAGAG |  |  |
| TSBP1-AS1 | rs3132966 | F: GGAATCTCCTGATCCACCAGTC | SR: | TTTTTTTTTTTTTTTTTTTTTTTGGGACTCATCAGGAAAACAACT |
|  |  | R: AGTAGGAAGAATCACACTATCCAATGT |  |  |
|  | rs6457513 | F: GGAATCTCCTGATCCACCAGTC | SF: | TTTTTTTTTTTTTTTTTTTTTTTTTTTTTTTTTTTAGCCATCTTGGCCTGGCAA |
|  |  | R: AGTAGGAAGAATCACACTATCCAATGT |  |  |
|  | rs9268000 | F: GGAATCTCCTGATCCACCAGTC | SR: | TTTTTTTTTTTTTTTTTTTTTTTTTTTTTTTTTTTTTTTTTTTTTTTCCAGCCAAGGGAAGAAGTGAG |
|  |  | R: AGTAGGAAGAATCACACTATCCAATGT |  |  |
| HCG9 | rs3823381 | F: CCAGCACAAAGGTGATTTTCTCC | SF: | TTTTTTTTTTTTTTTTTTTTTTTTTTTTTTTTTTTTTTTAGGACKGGGATCACCCATCA |
|  |  | R: CCGTTTCTATATCAGTAAACACTTGTCATT |  |  |

E, forward; R, reverse, SR, reverse gene-specific, SF, forward gene-specific.
