# Supplementary material for: Identification of rs28362336 as a risk SNP for generalized myasthenia gravis and its impact on HCG27 regulation
Source: PLoS One. 2026 Sep 23;21(9):e0359069. doi: 10.1371/journal.pone.0359069 (PMC13600526; doi:10.1371/journal.pone.0359069)
Supplement: Supplementary file 4 [file pone.0359069.s004.docx]

S3_Table. Feature Name

| Feature Type | Feature Name |
| --- | --- |
| Patient Demographics | Age |
|  | Sex |
| SNP | rs9263872 |
|  | rs9263875 |
|  | rs28362336 |
|  | rs3823381 |
| Blood Work | ALT |
| Feature Type | AST |
|  | GGT |
|  | TBIL |
|  | DBIL |
|  | Urea |
|  | Creatinine |
|  | Uric Acid |
|  | TG |
|  | HDL |
|  | LDL |
|  | WBC |
|  | Neutrophil Percentage |
|  | Lymphocyte Percentage |
|  | Monocyte Percentage |
|  | Eosinophil Percentage |
|  | Basophilic Count |
|  | Lymphocyte Count |
|  | Monocyte Count |
|  | Eosinophil Count |
|  | Hemoglobin |
|  | RBC |
|  | Hematocrit |
|  | Mean Corpuscular Hemoglobin |
|  | Mean Corpuscular Hemoglobin Concentration |
|  | Red Cell Distribution Width |
|  | Platelet Count |
|  | Mean Platelet Volume |
