# Supplementary material for: Identification of rs28362336 as a risk SNP for generalized myasthenia gravis and its impact on HCG27 regulation
Source: PLoS One. 2026 Sep 23;21(9):e0359069. doi: 10.1371/journal.pone.0359069 (PMC13600526; doi:10.1371/journal.pone.0359069)
Supplement: Supplementary file 5 [file pone.0359069.s005.docx]

S4_Table. Clinical features of RNA samples.

| Characteristic | MG  (n=35) | Control  (n=34) |
| --- | --- | --- |
| Age (y) | 61.83 ± 12.86 | 60.56 ± 11.29 |
| Gender (M/F) | 12/23 | 12/22 |
| EOMG (≤ 50 y) | 4 | - |
| LOMG (>50 y) | 31 | - |
| OMG | 22 | - |
| GMG | 13 | - |
| AChR Ab (Positive/Total) | 33/35 | - |

Among the 35 patients with MG, 33 patients had MG-related antibody test and 2

patients refused MG-related antibody test.
