# Supplementary material for: Identification of rs28362336 as a risk SNP for generalized myasthenia gravis and its impact on HCG27 regulation
Source: PLoS One. 2026 Sep 23;21(9):e0359069. doi: 10.1371/journal.pone.0359069 (PMC13600526; doi:10.1371/journal.pone.0359069)
Supplement: Supplementary file 6 [file pone.0359069.s006.docx]

S5_Table. The primers used for real‐time PCR

| Name |  | Sequences |
| --- | --- | --- |
| HCG27 | Forward primer | CACCTGTCTCCGAATCCGTC |
|  | Reverse primer | CTGTGAAAATGCAGACCCGC |
| HCG9 | Forward primer  Reverse primer | CCCACAGCAATAGGAAGCACCA  CCTGGAGTCCGCGTGTTCTCA |
| GAPDH | Forward primer | CATGTTCGTCATGGGTGTGAA |
|  | Reverse primer | GGCATGGACTGTGGTCATGAG |
