# Supplementary material for: Identification of rs28362336 as a risk SNP for generalized myasthenia gravis and its impact on HCG27 regulation
Source: PLoS One. 2026 Sep 23;21(9):e0359069. doi: 10.1371/journal.pone.0359069 (PMC13600526; doi:10.1371/journal.pone.0359069)
Supplement: Supplementary file 7 [file pone.0359069.s007.docx]

S6_Table. Twelve potentially functional SNP and their corresponding lncRNAs identified via database screening

| LncRNA | rs IDs | ReguLomeDB | | 3DSNP | | HaploReg | | | |
| --- | --- | --- | --- | --- | --- | --- | --- | --- | --- |
|  |  | score | QTL | Score | 3D  interacting  gene | Promoter histone marks | Enhancer histone marks | Proteins bound | Motif changed |
| LINC00243 | rs886423 | 1f | TRUE | 123.14 | FLOT1 and other8 genes | 17 | 42 | 15 | 4 |
|  | rs2894047 | 1a | TRUE | 86.43 | FLOT1 and other 8 genes | 4 | 19 | 4 | 3 |
| HCG27 | rs9263872 | 1b | TRUE | 81.06 | CCHCR1 and other 8 genes | 8 | 18 | 2 | 6 |
|  | rs9263875 | 1f | TRUE | 83.33 | CCHCR1 and other 8 genes | 5 | 18 | 1 | 3 |
|  | rs28362334 | 1b | TRUE | 206.32 | CCHCR1 and other 9 genes | 21 | 4 | 29 | 3 |
|  | rs28362336 | 1b | TRUE | 206.85 | CCHCR1 and other 9 genes | 21 | 4 | 31 | 6 |
|  | rs28362343 | 1a | TRUE | 215.06 | CCHCR1 and other 9 genes | 24 | 0 | 19 | 11 |
|  | rs6921948 | 1a | TRUE | 183.46 | CCHCR1 and other 8 genes | 15 | 15 | 20 | 14 |
| TSBP1-AS1 | rs3132966 | 1f | TRUE | 95.03 | ATF6B and other 20 genes | 16 | 11 | 2 | 6 |
|  | rs6457513 | 1f | TRUE | 92.92 | ATF6B and other 20 genes | 16 | 11 | 1 | 4 |
|  | rs9268000 | 1b | TRUE | 154.09 | ATF6B and other 20 genes | 20 | 12 | 4 | 4 |
| HCG9 | rs3823381 | 1b | TRUE | 212.85 | HLA-A and other 14 genes | 24 | 4 | 12 | 10 |
