# Supplementary material for: Identification of rs28362336 as a risk SNP for generalized myasthenia gravis and its impact on HCG27 regulation
Source: PLoS One. 2026 Sep 23;21(9):e0359069. doi: 10.1371/journal.pone.0359069 (PMC13600526; doi:10.1371/journal.pone.0359069)
Supplement: Supplementary file 8 [file pone.0359069.s008.docx]

S7_Table. Basic information of the included SNP loci

| LncRNA name | Rs number | Chrom | Position | Alleles |
| --- | --- | --- | --- | --- |
| LINC00243 | rs886423 | Chr6 | 30814428 | G>C |
| LINC00243 | rs2894047 | Chr6 | 30814630 | A>G |
| HCG27 | rs9263872 | Chr6 | 31202831 | C>T |
| HCG27 | rs9263875 | Chr6 | 31203137 | A>G |
| HCG27 | rs28362334 | Chr6 | 31197051 | C>A |
| HCG27 | rs28362336 | Chr6 | 31197124 | T>G |
| HCG27 | rs28362343 | Chr6 | 31197750 | G>A |
| HCG27 | rs6921948 | Chr6 | 31203480 | A>C |
| TSBP1-AS1 | rs3132966 | Chr6 | 32255855 | G>A |
| TSBP1-AS1 | rs6457513 | Chr6 | 32255993 | T>C |
| TSBP1-AS1 | rs9268000 | Chr6 | 32255754 | A>C |
| HCG9 | rs3823381 | Chr6 | 29976781 | C>T |
