# Supplementary material for: Identification of rs28362336 as a risk SNP for generalized myasthenia gravis and its impact on HCG27 regulation
Source: PLoS One. 2026 Sep 23;21(9):e0359069. doi: 10.1371/journal.pone.0359069 (PMC13600526; doi:10.1371/journal.pone.0359069)
Supplement: Supplementary file 9 [file pone.0359069.s009.docx]

S8_Table. Distribution of genotype and allele frequencies between the MG group and healthy controls

| SNP | | Genotype/Allele | | MG | | Control | | *P* for HWE | | χ^2^ | *P* | | OR (95%CI) |
| --- | --- | --- | --- | --- | --- | --- | --- | --- | --- | --- | --- | --- | --- |
|  |  |  |  | N(%) | | N(%) | |  |  |  |  |  |  |
| rs886423 | | GG | | 151(93.8) | | 151(93.8) | | 1 | | 0 | 1.000 | |  |
|  | | GC | | 10(6.2) | | 10(6.2) | |  | |  |  | | 1.000(0.405,2.472) |
|  | | G | | 312(96.9) | | 312(96.9) | | - | | 0 | 1.000 | |  |
|  | | C | | 10(3.1) | | 10(3.1) | |  | |  |  | | 1.000(0.410,2.436) |
| rs2894047 | | AA | | 26(16.1) | | 28(17.4) | | 0.3562 | |  |  | |  |
|  | | AG | | 84(52.2) | | 85(52.8) | |  | | 0.040 | 0.842 | | 1.064(0.576,1.965) |
|  | | GG | | 51(31.7) | | 48(29.8) | |  | | 0.158 | 0.691 | | 1.144(0.589,2.222) |
|  | | A | | 136(42.2) | | 141(43.8) | | - | | 0.158 | 0.691 | |  |
|  | | G | | 186(57.8) | | 181(56.2) | |  | |  |  | | 1.070(0.776,1.460) |
| rs9263872 | | CC | | 46(28.6) | | 43(26.7) | | 1 | |  |  | |  |
|  | | CT | | 77(47.8) | | 82(50.9) | |  | | 0.242 | 0.623 | | 0.878(0.522,1.475) |
|  | | TT | | 38(23.6) | | 36(22.4) | |  | | 0.002 | 0.966 | | 0.987(0.532,1.829) |
|  | | C | | 169(52.5) | | 168(52.2) | | - | |  | 0.937 | |  |
|  | | T | | 153(47.5) | | 154(47.8) | |  | | 0.006 |  | | 0.988(0.723,1.350) |
| rs9263875 | | AA | | 60(37.3) | | 57(35.4) | | 0.5082 | |  |  | |  |
|  | | GA | | 73(45.3) | | 75(46.6) | |  | | 0.100 | 0.752 | | 0.925(0.569,1.502) |
|  | | GG | | 28(17.4) | | 29(18.0) | |  | | 0.071 | 0.789 | | 0.917(0.487,1.728) |
|  | | A | | 193(59.9) | | 189(58.7) | | - | |  | 0.748 | |  |
|  | | G | | 129(40.1) | | 133(41.3) | |  | | 0.103 |  | | 0.950(0.697,1.290) |
| rs28362334 | | CC | | 138(85.7) | | 140(87.0) | | 1 | |  |  | |  |
|  | | CA | | 23(14.3) | | 21(13.0) | |  | | 0.105 | 0.746 | | 1.110(0.585,2.150) |
|  | | C | | 299(92.9) | | 301(93.5) | | - | | 0.098 | 0.877 | | 1.102(0.584,1.900) |
|  | | A | | 23(7.1) | | 21(6.5) | |  | |  |  | |  |
| rs28362336 | | TT | | 46(28.6) | | 46(28.6) | | 0.933 | |  |  | | - |
|  | | TG | | 80(49.7) | | 78(48.4) | |  | | 0.009 | 0.923 | 1.026(0.613,1.715) | |
|  | | GG | | 35(21.7) | | 37(23.0) | |  | | 0.031 | 0.86 | 0.946(0.510,1.753) | |
|  | | T | | 172(53.4) | | 170(52.8) | | - | |  | 0.875 |  | |
|  | | G | | 150(46.6) | | 152(47.2) | |  | | 0.025 |  | 0.975(0.714,1.330) | |
| rs28362343 | | GG | | 138(85.7) | | 140(87.0) | | 1 | |  |  | - | |
|  | | GA | | 23(14.3) | | 21(13.0) | |  | | 0.105 | 0.746 | 1.110(0.585,2.150) | |
|  | | G | | 299(92.9) | | 301(93.5) | | - | | 0.098 | 0.877 | 1.102(0.584,1.900) | |
|  | | A | | 23(7.1) | | 21(6.5) | |  | |  |  |  | |
| rs6921948 | | AA | | 65(40.4) | | 65(40.4) | | 0.9195 | |  |  |  | |
|  | | AC | | 74(46.0) | | 74(46.0) | |  | | **-** | 1.000 | 1.000(0.624,1.602) | |
|  | | CC | | 22(13.7) | | 22(13.7) | |  | | **-** | 1.000 | 1.000(0.505,1.981) | |
|  | | A | | 204(63.4) | | 204(63.4) | | - | |  |  |  | |
|  | | C | | 118(36.6) | | 118(36.6) | |  | | **-** | 1.000 | 1.000(0.726,1.378) | |
| rs3132966 | | GG | | 102(63.4) | | 112(69.6) | | 0.368 | |  |  |  | |
|  | | GA | | 56(34.8) | | 44(27.3) | |  | | 1.895 | 0.169 | 1.398(0.867,2.252) | |
|  | | AA | | 3(1.9) | | 5(3.1) | |  | | 0.32 | 0.838 | 0.659(0.154,2.826) | |
|  | | G | | 260(80.7) | | 268(83.2) | | - | |  | 0.412 |  | |
|  | | A | | 62(19.3) | | 54(16.8) | |  | | 0.673 |  | 1.183(0.793,1.780) | |
| rs6457513 | | TT | | 101(62.7) | | 110(68.3) | | 0.2741 | |  |  |  | |
|  | | TC | | 57(35.4) | | 46(28.6) | |  | | 1.546 | 0.214 | 1.350(0.841,2.166) | |
|  | | CC | | 3(1.9) | | 5(3.1) | |  | | 0.332 | 0.562 | 0.653(0.152,2.804) | |
|  | | T | | 259(80.4) | | 266(82.6) | |  | |  |  |  | |
|  | | C | | 63(19.6) | | 56(17.4) | |  | | 0.505 | 0.477 | 1.155(0.780,1.722) | |
| rs9268000 | | AA | | 65(40.4) | | 60(37.3) | | 0.0534 | |  |  |  | |
|  | AC | | 83(51.6) | | 81(50.3) | |  | | 0.055 | | 0.815 | | 0.946(0.594,1.507) |
|  | CC | | 13(8.1) | | 20(12.4) | |  | | 1.660 | | 0.198 | | 0.600(0.275,1.311) |
|  | A | | 213(66.1) | | 201(62.4) | |  | |  | |  | |  |
|  | C | | 109(33.9) | | 121(37.6) | |  | | 0.974 | | 0.324 | | 0.850(0.613,1.180) |
| rs3823381 | CC | | 50(31.1) | | 57(35.4) | | 1 | |  | |  | |  |
|  | CT | | 85(52.8) | | 70(43.5) | |  | | 1.667 | | 0.197 | | 1.384(0.844,2.269) |
|  | TT | | 26(16.1) | | 34(21.1) | |  | | 0.179 | | 0.672 | | 0.872(0.461,1.647) |
|  | C | | 185(57.5) | | 184(57.1) | | - | |  | |  | |  |
|  | T | | 137(42.5) | | 138(42.9) | |  | | 0.006 | | 0.937 | | 0.987(0.719,1.357) |

OR, odds ratio; 95 % CI, 95 % confidence interval. The reference group is the HC group.
