# Supplementary material for: Identification of rs28362336 as a risk SNP for generalized myasthenia gravis and its impact on HCG27 regulation
Source: PLoS One. 2026 Sep 23;21(9):e0359069. doi: 10.1371/journal.pone.0359069 (PMC13600526; doi:10.1371/journal.pone.0359069)
Supplement: Supplementary file 10 [file pone.0359069.s011.docx]

S10_Table. Distribution of SNP genotype and allele frequencies in groups stratified by age

| SNP | | Genotype/Allele | | | Age | | | | | | χ^2^ | | | *P* | | OR (95%CI) |
| --- | --- | --- | --- | --- | --- | --- | --- | --- | --- | --- | --- | --- | --- | --- | --- | --- |
|  |  |  |  |  | ＞50 years | | | ≤50 years | | |  |  |  |  |  |  |
| rs886423 | | GG | | | 124(93.2) | | | 27(96.4) | | |  | | |  | |  |
|  | | GC | | | 9(6.8) | | | 1(3.6) | | | **-** | | | 1.000 | | 0.519(0.062,4.199) |
|  | | | G | | | 257(96.6) | | | 55(98.2) | | |  | | |  |  |
|  | | | C | | | 9(3.4) | | | 1(1.8) | | | **-** | | | 1.000 | 0.733(0.128,4.200) |
| rs2894047 | | | AA | | | 20(15.0) | | | 6(21.4) | | |  | | |  |  |
|  | | | AG | | | 68(51.1) | | | 16(57.1) | | | 0.201 | | | 0.654 | 0.784(0.271,2.323) |
|  | | | GG | | | 45(33.8) | | | 6(21.4) | | | 1.675 | | | 0.196 | 0.444(0.132,1.611) |
|  | | | A | | | 108(40.6) | | | 28(50.0) | | |  | | |  |  |
|  | | | G | | | 158(59.4) | | | 28(50.0) | | | 1.675 | | | 0.196 | 0.684(0.383,1.219) |
| rs9263872 | | | CC | | | 37(27.8) | | | 9(32.1) | | |  | | |  |  |
|  | | | CT | | | 64(48.1) | | | 13(46.4) | | | 0.141 | | | 0.707 | 0.835(0.338,2.011) |
|  | | | TT | | | 32(24.1) | | | 6(21.4) | | | 0.202 | | | 0.653 | 0.771(0.235,2.458) |
|  | | | C | | | 138(51.9) | | | 31(55.4) | | |  | | |  |  |
|  | | | T | | | 128(48.1) | | | 25(44.6) | | | 0.224 | | | 0.636 | 0.869(0.487,1.552) |
| rs9263875 | | | AA | | | 48(36.1) | | | 12(42.9) | | |  | | |  |  |
|  | | | GA | | | 61(45.9) | | | 12(42.9) | | | 0.282 | | | 0.595 | 0.787(0.340,1.824) |
|  | | | GG | | | 24(18.0) | | | 4(14.3) | | | - | | | 0.517 | 0.667(0.218,2.193) |
|  | | | A | | | 157(59.0) | | | 36(64.3) | | |  | | |  |  |
|  | | | G | | | 109(41.0) | | | 20(35.7) | | | 0.534 | | | 0.465 | 0.800(0.440,1.456) |
| rs28362334 | | | CC | | | 114(85.7) | | | 24(85.7) | | |  | | |  |  |
|  | | | CA | | | 19(14.3) | | | 4(14.3) | | | - | | | 1.000 | 1.000(0.312,3.205) |
|  | | | C | | | 247(92.9) | | | 52(92.9) | | |  | | |  |  |
|  | | | A | | | 19(7.1) | | | 4(7.1) | | | - | | | 1.000 | 1.000(0.356,3.063) |
| rs28362336 | | | TT | | | 37(27.8) | | | 9(32.1) | | |  | | |  |  |
|  | | | TG | | | 67(50.4) | | | 13(46.4) | | | 0.223 | | | 0.637 | 0.798(0.324,1.916) |
|  | | | GG | | | 29(21.8) | | | 6(21.4) | | | 0.077 | | | 0.781 | 0.851(0.257,2.744) |
|  | | | T | | | 141(53.0) | | | 31(55.4) | | |  | | |  |  |
|  | | | G | | | 125(47.0) | | | 25(44.6) | | | 0.103 | | | 0.749 | 0.910(0.510,1.624) |
| rs28362343 | | | GG | | | 114(85.7) | | | 24(85.7) | | |  | | |  |  |
|  | | | GA | | | 19(14.3) | | | 4(14.3) | | | - | | | 1.000 | 1.000(0.312,3.205) |
|  | | | G | | | 247(92.9) | | | 52(92.9) | | |  | | |  |  |
|  | | | A | | | 19(7.1) | | | 4(7.1) | | | - | | | 1.000 | 1.000(0.356,3.063) |
| rs6921948 | | | AA | | | 54(40.6) | | | 11(39.3) | | |  | | |  |  |
|  | | | AC | | | 61(45.9) | | | 13(46.4) | | | 0.010 | | | 0.92 | 1.046(0.444,2.477) |
|  | | | CC | | | 18(13.5) | | | 4(14.3) | | | - | | | 1.000 | 1.091(0.347,3.899) |
|  | | | A | | | 169(63.5) | | | 35(62.5) | | |  | | |  |  |
|  | | | C | | | 97(36.5) | | | 21(37.5) | | | 0.021 | | | 0.884 | 1.045(0.576,1.897) |
| rs3132966 | | | GG | | | 85(63.9) | | | 17(60.7) | | |  | | |  |  |
|  | | | GA | | | 45(33.8) | | | 11(39.3) | | | 0.220 | | | 0.639 | 1.222(0.528,2.831) |
|  | | | AA | | | 3(2.3) | | | 0(0) | | | - | | | 1.000 | - |
|  | | | G | | | 215(80.8) | | | 45(80.4) | | |  | | |  |  |
|  | | | A | | | 51(19.2) | | | 11(19.6) | | | 0.007 | | | 0.935 | 1.031(0.498,2.131) |
| rs6457513 | | | TT | | | 84(63.2) | | | 17(60.7) | | |  | | |  |  |
|  | | | TC | | | 46(34.6) | | | 11(39.3) | | | 0.152 | | | 0.697 | 1.182(0.510,2.735) |
|  | | | CC | | | 3(2.3) | | | 0(0) | | | - | | | 1.000 | - |
|  | | | T | | | 214(80.5) | | | 45(80.4) | | |  | | |  |  |
|  | | | C | | | 52(19.5) | | | 11(19.6) | | | - | | | 0.987 | 1.006(0.487,2.078) |
| rs9268000 | | | AA | | | 52(39.1) | | | 13(46.4) | | |  | | |  |  |
|  | | | AC | | | 71(53.4) | | | 12(42.9) | | | 0.798 | | | 0.372 | 0.676(0.302,1.660) |
|  | | | CC | | | 10(7.5) | | | 3(10.7) | | | - | | | 0.723 | 1.200(0.317,4.597) |
|  | | | A | | | 175(65.8) | | | 38(67.9) | | |  | | |  |  |
|  | | | C | | | 91(34.2) | | | 18(32.1) | | | 0.088 | | | 0.766 | 0.911(0.492,1.686) |
| rs3823381 | | | CC | | | 43(32.3) | | | 7(25.0) | | |  | | |  |  |
|  | | | CT | | | 69(51.9) | | | 16(57.1) | | | 0.518 | | | 0.472 | 1.424(0.526,3.616) |
|  | TT | | | 21(15.8) | | | 5(17.9) | | | 0.352 | | | 0.553 | | | 1.463(0.450,5.071) |
|  | C | | | 155(58.3) | | | 30(53.6) | | |  | | |  | | |  |
|  | T | | | 111(41.7) | | | 26(46.4) | | | 0.418 | | | 0.518 | | | 1.210(0.678,2.159) |

OR, odds ratio; 95 % CI, 95 % confidence interval. The reference group consists of individuals over the age of 50.
