# Supplementary material for: Identification of rs28362336 as a risk SNP for generalized myasthenia gravis and its impact on HCG27 regulation
Source: PLoS One. 2026 Sep 23;21(9):e0359069. doi: 10.1371/journal.pone.0359069 (PMC13600526; doi:10.1371/journal.pone.0359069)
Supplement: Supplementary file 11 [file pone.0359069.s013.docx]

S12_Table. Distribution of SNP genotype and allele frequencies in groups stratified by gender

| SNP | Genotype/Allele | | Sex | | | | χ^2^ | | *P* | OR (95%CI) |
| --- | --- | --- | --- | --- | --- | --- | --- | --- | --- | --- |
|  |  |  | Male | | Female | |  |  |  |  |
| rs886423 | GG | | 62(92.5) | | 89(94.7) | |  | |  |  |
|  | GC | | 5(7.5) | | 5(5.3) | | 0.309 | | 0.579 | 0.697(0.193,2.509) |
|  | G | | 129(96.3) | | 183(97.3) | |  | |  |  |
|  | C | | 5(3.7) | | 5(2.7) | | 0.299 | | 0.585 | 0.705(0.200,2.485) |
| rs2894047 | AA | | 11(16.4) | | 15(16.0) | |  | |  |  |
|  | AG | | 36(53.7) | | 48(51.1) | | 0.002 | | 0.961 | 0.978(0.405,2.452) |
|  | GG | | 20(29.9) | | 31(33.0) | | 0.068 | | 0.794 | 1.137(0.414,2.950) |
|  | A | | 58(43.3) | | 78(41.5) | |  | |  |  |
|  | G | | 76(56.7) | | 110(58.5) | | 0.103 | | 0.748 | 1.076(0.687,1.685) |
| rs9263872 | CC | | 20(29.9) | | 26(27.7) | |  | |  |  |
|  | | CT | | 30(44.8) | | 47(50.0) | | 0.244 | 0.622 | 1.205(0.582,2.460) |
|  | | TT | | 17(25.4) | | 21(22.3) | | 0.013 | 0.908 | 0.950(0.393,2.312) |
|  | | C | | 70(52.2) | | 99(52.7) | |  |  |  |
|  | | T | | 64(47.8) | | 89(47.3) | | 0.006 | 0.941 | 0.983(0.631,1.532) |
| rs9263875 | | AA | | 23(34.3) | | 37(39.4) | |  |  |  |
|  | | GA | | 31(46.3) | | 42(44.7) | | 0.233 | 0.629 | 0.842(0.413,1.680) |
|  | | GG | | 13(19.4) | | 15(16.0) | | 0.518 | 0.472 | 0.717(0.304,1.723) |
|  | | A | | 77(57.5) | | 116(61.7) | |  |  |  |
|  | | G | | 57(42.5) | | 72(38.3) | | 0.586 | 0.444 | 0.838(0.534,1.317) |
| rs28362334 | | CC | | 60(89.6) | | 78(83.0) | |  |  |  |
|  | | CA | | 7(10.4) | | 16(17.0) | | 1.380 | 0.240 | 1.758(0.680,4.546) |
|  | | C | | 127(94.8) | | 172(91.5) | |  |  |  |
|  | | A | | 7(5.2) | | 16(8.5) | | 1.274 | 0.259 | 1.688(0.674,4.224) |
| rs28362336 | | TT | | 20(29.9) | | 26(27.7) | |  |  |  |
|  | | TG | | 30(44.8) | | 50(53.2) | | 0.436 | 0.509 | 1.282(0.623,2.601) |
|  | | GG | | 17(25.4) | | 18(19.1) | | 0.208 | 0.649 | 0.814(0.322,2.042) |
|  | | T | | 70(52.2) | | 102(54.3) | |  |  |  |
|  | | G | | 64(47.8) | | 86(45.7) | | 0.128 | 0.721 | 0.922(0.591,1.438) |
| rs28362343 | | GG | | 60(89.6) | | 78(83.0) | |  |  |  |
|  | | GA | | 7(10.4) | | 16(17.0) | | 1.380 | 0.240 | 1.758(0.680,4.546) |
|  | | G | | 127(94.8) | | 172(91.5) | |  |  |  |
|  | | A | | 7(5.2) | | 16(8.5) | | 1.274 | 0.259 | 1.688(0.674,4.224) |
| rs6921948 | | AA | | 25(37.3) | | 40(42.6) | |  |  |  |
|  | | AC | | 31(46.3) | | 43(45.7) | | 0.169 | 0.681 | 0.867(0.442,1.676) |
|  | | CC | | 11(16.4) | | 11(11.7) | | 0.902 | 0.342 | 0.625(0.235,1.737) |
|  | | A | | 81(60.4) | | 123(65.4) | |  |  |  |
|  | C | | 53(39.6) | | 65(34.6) | | 0.835 | | 0.361 | 0.808(0.511,1.277) |
| rs3132966 | GG | | 46(68.7) | | 56(59.6) | |  | |  |  |
|  | GA | | 21(31.3) | | 35(37.2) | | 0.855 | | 0.355 | 1.369(0.703,2.667) |
|  | AA | | 0(0) | | 3(3.2) | | - | | 0.254 | - |
|  | G | | 113(84.3) | | 147(78.2) | |  | |  |  |
|  | A | | 21(15.7) | | 41(21.8) | | 1.895 | | 0.169 | 1.501(0.840,2.681) |
| rs6457513 | TT | | 46(68.7) | | 55(58.5) | |  | |  |  |
|  | TC | | 21(31.3) | | 36(38.3) | | 1.130 | | 0.288 | 1.434(0.737,2.790) |
|  | CC | | 0(0) | | 3(3.2) | | - | | 0.253 | - |
|  | T | | 113(84.3) | | 146(77.7) | |  | |  |  |
|  | C | | 21(15.7) | | 42(22.3) | | 2.211 | | 0.137 | 1.548(0.868,2.761) |
| rs9268000 | AA | | 22(32.8) | | 43(45.7) | |  | |  |  |
|  | AC | | 39(58.2) | | 44(46.8) | | 2.598 | | 0.107 | 0.577(0.296,1.145) |
|  | CC | | 6(9.0) | | 7(7.4) | | 0.713 | | 0.398 | 0.597(0.185,2.176) |
|  | A | | 83(61.9) | | 130(69.1) | |  | |  |  |
|  | C | | 51(38.1) | | 58(30.9) | | 1.816 | | 0.178 | 0.726(0.456,1.157) |
| rs3823381 | CC | | 25(37.3) | | 25(26.6) | |  | |  |  |
|  | CT | | 37(55.2) | | 48(51.1) | | 0.531 | | 0.466 | 1.297(0.640,2.644) |
|  | TT | | 5(7.5) | | 21(22.3) | | 6.778 | | 0.009 | 4.200(1.369,11.280) |
|  | C | | 87(64.9) | | 98(52.1) | |  | |  |  |
|  | T | | 47(35.1) | | 90(47.9) | | 5.242 | | 0.022 | 1.700(1.078,2.682) |

OR, odds ratio; 95 % CI, 95 % confidence interval. The reference group is the male group.
